# Protein-energy malnutrition worsens hospitalization outcomes of patients with pancreatic cancer undergoing open pancreaticoduodenectomy

**DOI:** 10.1007/s13304-022-01293-7

**Published:** 2022-05-07

**Authors:** Yu-Xiu Zhang, Yi-Feng Yang, Pu Han, Peng-Cheng Ye, Hao Kong

**Affiliations:** 1 Department of Anesthesiology and Critical Care Medicine, Peking University First Hospital, No. 8 Xishiku Street, Beijing, 100034 China; 2 Department of Internal Medicine, University of Iowa Hospitals and Clinic, Iowa, USA; 3 Department of General Surgery, Beijing Key Laboratory of Cancer Invasion and Metastasis Research & National Clinical Research Center for Digestive Diseases, Beijing Friendship Hospital, Capital Medical University, 95 Yong-an Road, Xi-Cheng District, Beijing, 100050 China

**Keywords:** Pancreatic cancer, Pancreaticoduodenectomy, Protein-energy malnutrition, Complication

## Abstract

To assess the role of protein-energy malnutrition on perioperative outcomes in patients with pancreatic cancer undergoing open pancreaticoduodenectomy. We conducted a retrospective observational cohort study and investigated patients ≥ 18 years old with pancreatic cancer undergoing open pancreaticoduodenectomy within the National inpatient sample database during 2012–2014. The study population was divided into two groups based on the presence of protein-energy malnutrition. In-hospital mortality, length of stay, cost of hospitalization, and in-hospital complications were compared between the two groups. Logistic and linear regression analyses were used to adjust for potential confounders. A trend analysis was further conducted on the in-hospital outcomes. Of the 12,785 patients aged ≥ 18 years undergoing open pancreaticoduodenectomy during years 2012–2014, 9865 (77.0%) had no protein-energy malnutrition and 2920 (23.0%) had protein-energy malnutrition. Patients with protein-energy malnutrition were found to have significantly higher mortality rate, longer length of hospital stay, and higher total hospital cost compared to those without protein-energy malnutrition. The risks of gastroparesis, small bowel obstruction, intraoperative and postoperative hemorrhage, infectious complications, and several systemic complications were found to be significantly higher in the protein-energy malnutrition group in a multivariate regression model. A study of trends from 2009 to 2012 revealed an increasing prevalence of protein-energy malnutrition, a declining trend in mortality and length of stay and a stable total hospital cost in the protein-energy malnutrition group. Protein-energy malnutrition was found to be associated with higher mortality, longer length of hospital stay and greater hospital cost in pancreatic cancer patients undergoing open pancreaticoduodenectomy, as well as increased occurrence of various systemic complications. Attention should be paid to patients’ nutritional status, which can be corrected before surgery as an effective means to optimize postoperative results.

## Background

Pancreatic cancer is the fourth most common cause of cancer death in the United States [1]. The 5-year survival rate is 10% at the time of diagnosis and 20% for patients who have undergone surgery for a resectable tumor [1, 2]. Pancreaticoduodenectomy (PD), also known as the Whipple procedure, proposed by Whipple et al. in 1935, is the reference treatment modality for resectable pancreatic cancer [3]. The resection extent of PD covers the duodenum, the proximal 15 cm of the jejunum, the common bile duct, gall bladder, head of the pancreas, and a distal gastrectomy [4]. According to the literature, the incidence of postoperative complications of PD is 27.1% or even higher [5].

Protein-energy malnutrition (PEM) is a state caused by inadequate intake of nutrition, which will lead to altered body composition resulting in physical and mental function decline [6]. The cause of PEM is multifactorial and, consists of starvation, disease or advanced ageing, alone or in combination [7]. Moreover, PEM is associated with a higher incidence of poor prognosis [8, 9]. Therefore, exploring the potential impact of PEM on the prognosis of patients receiving open pancreaticoduodenectomy (OPD) is of great significance, as it can stimulate targeted interventions and improve prognosis. According to the literature in gastrointestinal (GI) cancer surgery, [10] vascular surgery, [11] cardiac surgery [12] and retroperitoneal sarcoma surgery, [13] patients with PEM are more likely to develop postoperative complications. However, there are few studies that focus on the influence of PEM on OPD prognosis and postoperative complications, especially those from a national cohort.

To this end, this study intends to reveal the impact of PEM on the hospitalization outcomes of patients receiving OPD through the analysis of nationwide data.

## Methods

### Study design

We conducted a retrospective observational cohort study and investigated patients ≥ 18 years old with pancreatic cancer undergoing OPD in the National inpatient sample (NIS) database during 2012–2014. The study was reported in accordance with Strengthening the Reporting of Observational Studies in Epidemiology (STROBE) guideline. Since the information in the database has been de-identified, this study is exempt from institutional review board evaluation.

### Study population

The NIS is supported by the Healthcare Cost and Utilization Project and is one of the largest inpatient administrative databases in the United States. Data are collected on nearly 8 million hospital discharges from more than 1000 hospitals per year and represent approximately 20% of hospitalization in the United States.

The International classification of diseases, ninth revision, clinical modification (ICD-9-CM) codes were used to identify pancreatic cancer patients (157) who underwent OPD (procedure code 52.7 was included, while 17.4 and 54.21 were excluded). The patients were then categorized into two groups: with and without PEM (260, 261, 262, 263.x[263.0, 263.1, 263.2, 263.8, 263.9], 799.4, 783.3, 269.8, 783.21, 783.22, 783.7) [9, 14, 15].

Patient characteristics included gender, race (White, Black, Hispanic, Asian or Pacific Islander, Native American and other), age, annual income, and insurance type (Medicare, Medicaid, private insurance, and others). Hospital characteristics included hospital region (northeast, midwest, south and west), hospital bed size (small, medium, and large), hospital location (rural and urban) and teaching status. Patient and hospital characteristics were coded as per NIS guidelines.

Twenty-three comorbid conditions across all the systems of the body were identified and the Charlson comorbidity index was used to summarize these comorbid factors [16, 17].

### Study outcomes

A total of 32 outcomes were studied: three primary and 29 secondary outcomes. The primary outcomes included a composite endpoint of major postoperative complications, including mortality, length of stay (LOS), and total cost. Secondary outcomes included surgery-related complications and associated systemic complications. In-hospital mortality, total cost and LOS were variables already present in the NIS, and other outcome variables were identified through ICD-9-CM.

### Statistical analysis

Stratification, clustering, and weighting were applied during the analysis to accommodate the NIS design. In our model, we used statements “egen STA = group (YEAR NIS_STRATUM)” and “svyset [pweight = DISCWT], strata (STA) psu (HOSP_NIS).” The stratum statement specifies NIS_STRATUM as the stratum identifier, and the cluster statement specifies HOSP_NIS as the cluster identifier.

We used a survey‐specific method, with the commands svyset and svy with pweight using the discharge‐level weight from 2012 to 2014 to generate nationwide estimates.

Baseline characteristics were compared between the two groups. Categorical variables were compared using the Chi-square test, and continuous variables were compared using a linear regression model.

Multivariable regression analysis was used to adjust for potential confounders, including demographics, hospital characteristics, and comorbidities. We assessed differences in binary outcomes using logistic regression and in continuous outcomes using ordinary least squares linear regression. We also conducted a trend study. Analyses were performed by Stata version 14 (College Station, TX: StataCorp LP). All *P* values were two-sided, with 0.05 as the threshold for statistical significance.

## Results

### Baseline characteristics

**Fig. 1:**
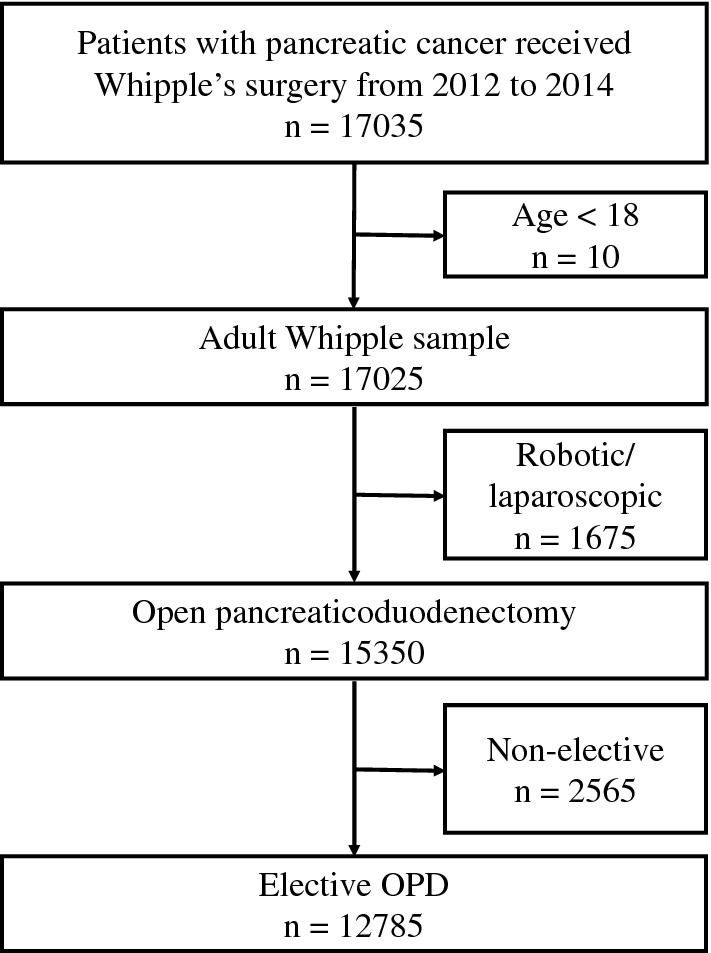
Patient identification flow diagram

A total of 12,785 patients aged ≥ 18 years underwent OPD during 2012–2014 (Fig. 1). Within this population, 2920 (23.0%) had a clinical diagnosis for PEM versus 9865 (77.0%) who did not.

Those with PEM were older, with a mean age of 67.4 years compared with 65.3 in the non-PEM group (*P* < 0.001). Compared to the non-PEM group, more patients in the PEM group came from low-income areas (*P* = 0.012) and there were significantly more Medicaid patients (*P* < 0.001). No statistically significant differences were found in gender, race, hospital region, bed size, location or teaching status.

**Fig. 2:**
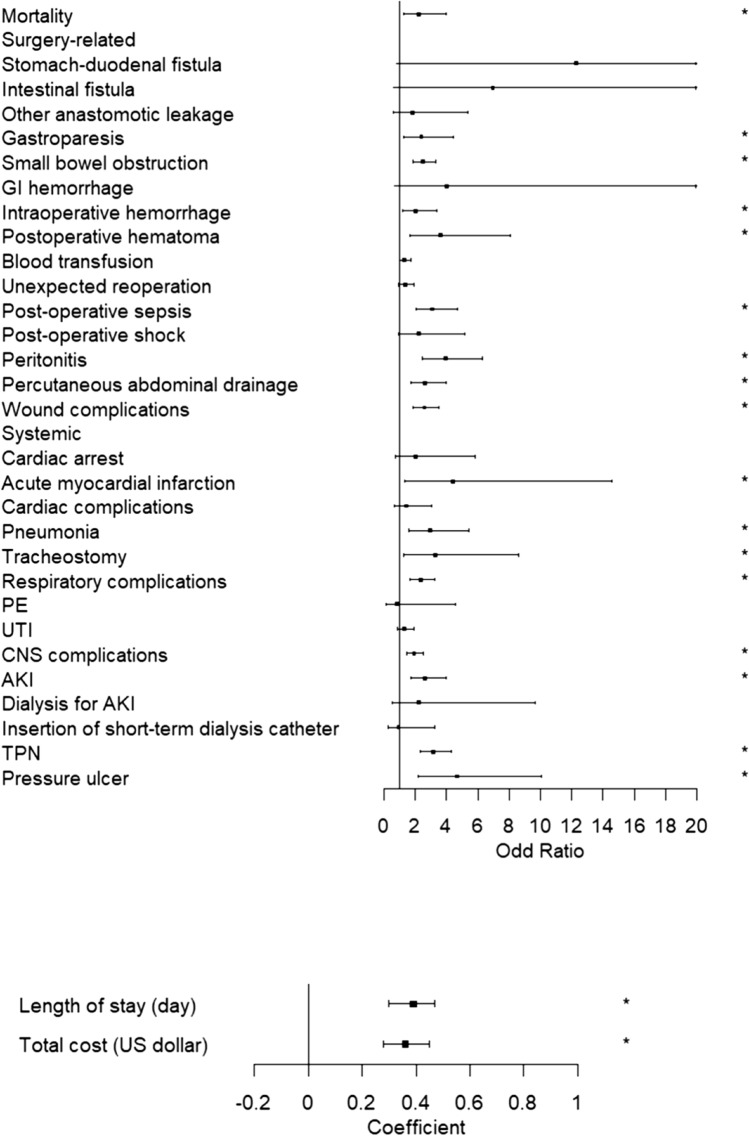
Forest plot of in-hospital outcomes

As for comorbidities, patients with PEM had a higher prevalence of coronary artery disease, previous myocardial infarction, peripheral vascular disease, chronic kidney disease, thrombocytopenia and anemia. Table 1 summarizes the results of the patients’ characteristics and comorbidities.

**Table 1:** Characteristics of included patients

	No PEM	PEM	*P* value
Patient characteristics			
No. (%) of patients	9865 (77.00%)	2920 (23.00%)	
Female	4693 (47.44%)	1286 (44.50%)	0.205
Race			0.330
White	7620 (77.19%)	2320 (79.63%)	
Black	807 (8.18%)	273 (9.36%)	
Hispanic	738 (7.48%)	171 (5.87%)	
Asian or Pacific Islander	337 (3.41%)	59 (2.02%)	
Native American	37 (0.38%)	5 (0.18%)	
Other	332 (3.36%)	86 (2.94%)	
Median age, y	65.3	67.4	< 0.001
Median annual income in patient’s zip code, US$, no. (%)			0.012
$1—$38,999	1932 (19.64%)	714 (24.22%)	
$39,000—$47,999	2355 (23.94%)	770 (26.12%)	
$48,000—$62,999	2595 (26.37%)	760 (25.78%)	
$63,000 or more	2957 (30.05%)	703 (23.88%)	
Insurance type, no. (%)			< 0.001
Medicaid	5159 (52.37%)	1888 (64.34%)	
Medicare	642 (6.51%)	164 (5.59%)	
Private	3880 (39.40%)	837 (28.50%)	
Uninsured	169 (1.72%)	46 (1.57%)	
Hospital characteristics			
Hospital region, no. (%)			0.157
Northeast	2230 (22.61%)	535 (18.32%)	
Midwest	2040 (20.68%)	735 (25.17%)	
South	3630 (36.80%)	1060 (36.30%)	
West	1965 (19.92%)	590 (20.21%)	
Hospital bed size, no. (%)			0.234
Small	855 (8.67%)	185 (6.34%)	
Medium	1830 (18.55%)	565 (19.35%)	
Large	7180 (72.78%)	2170 (74.32%)	
Location of hospital			0.852
Rural hospital	75 (0.76%)	25 (0.86%)	
Urban hospital	9789 (99.24%)	2895 (99.14%)	
Teaching status of hospital			0.172
Rural and urban non-teaching hospital	900 (9.12%)	325 (11.13%)	
Urban teaching hospital	8965 (90.88%)	2595 (88.87%)	
Comorbidities			
Hypertension	5775 (58.54%)	1714 (58.73%)	0.934
Diabetes mellitus	3245 (32.89%)	1035 (35.45%)	0.261
Obesity	1060 (10.75%)	275 (9.42%)	0.333
Smoking	3410 (34.47%)	896 (31.01%)	0.129
Alcohol use	206 (2.08%)	85 (2.95%)	0.209
Drug use	1363 (13.77%)	470 (16.28%)	0.123
AIDS	4 (0.05%)	9 (0.31%)	0.068
Coronary artery disease	1255 (12.72%)	545 (18.66%)	< 0.001
Congestive heart failure	270 (2.74%)	125 (4.28%)	0.066
History of myocardial infarction	360 (3.65%)	185 (6.34%)	0.005
Arrhythmia	2254 (22.78%)	753 (26.05%)	0.102
Valvular heart disease	325 (3.29%)	70 (2.40%)	0.257
Peripheral vascular disease	315 (3.19%)	165 (5.65%)	0.004
Chronic obstructive lung disease	700 (7.10%)	270 (9.25%)	0.077
Paresis	27 (0.27%)	13 (0.47%)	0.442
History of stroke	65 (0.66%)	15 (0.51%)	0.700
Hyperthyroidism	27 (0.27%)	13 (0.47%)	0.439
Hypothyroidism	1183 (11.96%)	332 (11.47%)	0.738
Chronic kidney disease	350 (3.55%)	250 (8.56%)	< 0.001
Thrombocytopenia	330 (3.35%)	205 (7.02%)	< 0.001
Lymphoma	45 (0.45%)	4 (0.16%)	0.284
Anemia	1175 (11.91%)	700 (23.97%)	< 0.001
Chronic liver disease	1225 (12.42%)	325 (11.13%)	0.390
Charlson score			N/A
0	0 (0%)	0 (0%)	
1	0 (0%)	0 (0%)	
2	3261 (32.42%)	865 (31.73%)	
> = 3	6799 (67.58%)	1860 (68.27%)	

*AID*S acquired immunodeficiency syndrome

### Primary outcomes

After adjustment for patient and hospital characteristics, hospitalized patients with PEM who underwent OPD had a 1.15-fold higher risk of in-hospital mortality (5.49% vs 2.48%, *P* = *0.005*) in comparison to those without PEM.

Multivariate linear regression analysis showed that patients with PEM had a longer hospital stay (16.4 vs 10.8 days, *P* < 0.001) and higher total hospital cost than those without PEM (53,595.71 USD vs 36,614.19, *P* < 0.001; Table 2 and Fig. 2).

**Table 2:** In-hospital outcomes

In-Hospital Outcomes	No PEM	PEM	Adjusted odds ratio	*P* value
Mortality	2.48%	5.49%	2.25 (1.28–3.96)	0.005
Length of stay (coefficient)	10.8	16.4	0.39 (0.3–0.47)	< 0.001
Total cost (coefficient)	36,614.19	53,595.71	0.36 (0.28–0.45)	< 0.001
Surgery-related				
Stomach-duodenal fistula	13 (0.14%)	27 (0.93%)	12.28 (0.79–190.03)	0.073
Intestinal fistula	9 (0.09%)	22 (0.78%)	6.96 (0.63–76.78)	0.113
Other anastomotic leakage	67 (0.68%)	40 (1.4%)	1.81 (0.61–5.35)	0.285
Gastroparesis	270 (2.74%)	180 (6.16%)	2.39 (1.28–4.47)	0.006
Small bowel obstruction	1067 (10.78%)	645 (22.33%)	2.48 (1.88–3.29)	< 0.001
GI hemorrhage	30 (0.3%)	15 (0.51%)	4.04 (0.67–24.45)	0.129
Intraoperative hemorrhage	282 (2.85%)	139 (4.81%)	2.01 (1.18–3.41)	0.010
Postoperative hematoma	166 (1.68%)	103 (3.57%)	3.64 (1.64–8.11)	0.002
Blood transfusion	1960 (19.87%)	725 (24.83%)	1.31 (0.99–1.74)	0.062
Unexpected reoperation	959 (9.69%)	412 (14.26%)	1.36 (0.97–1.91)	0.073
Post-operative sepsis	380 (3.85%)	395 (13.53%)	3.1 (2.04–4.7)	< 0.001
Post-operative shock	121 (1.24%)	106 (3.54%)	2.21 (0.94–5.16)	0.068
Peritonitis	287 (2.9%)	336 (11.63%)	3.96 (2.49–6.31)	< 0.001
Percutaneous abdominal drainage	399 (4.03%)	314 (10.85%)	2.62 (1.73–3.98)	< 0.001
Wound complications	1210 (12.27%)	780 (26.71%)	2.59 (1.89–3.55)	< 0.001
Systemic				
Cardiac arrest	85 (0.86%)	60 (2.05%)	2.05 (0.72–5.82)	0.179
Acute myocardial infarction	45 (0.46%)	55 (1.88%)	4.39 (1.32–14.59)	0.016
Cardiac complications	220 (2.23%)	80 (2.74%)	1.45 (0.69–3.07)	0.330
Pneumonia	225 (2.28%)	190 (6.51%)	2.95 (1.6–5.43)	0.001
Tracheostomy	54 (0.54%)	72 (2.48%)	3.27 (1.24–8.6)	0.017
Respiratory complications	840 (8.51%)	535 (18.32%)	2.34 (1.69–3.25)	< 0.001
PE	60 (0.61%)	20 (0.68%)	0.85 (0.16–4.56)	0.850
UTI	620 (6.28%)	280 (9.59%)	1.28 (0.84–1.94)	0.251
CNS complications	1372 (13.86%)	686 (23.72%)	1.93 (1.46–2.54)	< 0.001
AKI	565 (5.73%)	475 (16.27%)	2.63 (1.74–3.97)	< 0.001
Dialysis for AKI	40 (0.41%)	45 (1.55%)	2.21 (0.51–9.68)	0.292
Insertion of short-term dialysis catheter	67 (0.68%)	45 (1.55%)	0.94 (0.27–3.26)	0.923
TPN	816 (8.24%)	650 (22.48%)	3.18 (2.33–4.33)	< 0.001
Pressure ulcer	54 (0.54%)	94 (3.26%)	4.68 (2.18–10.07)	< 0.001

*GI* gastrointestinal, *AKI*, acute kidney injury, *PE* pulmonary embolism, *UTI* urinary tract infection, *CNS* central nervous system, *TPN* total parenteral nutrition

### Secondary outcomes

As shown in Table 2 and Fig. 2, PEM correlated with higher odds for gastroparesis (6.16% vs 2.74%, *P* = 0.006), small bowel obstruction (22.33% vs 10.78%, *P* < *0.001*), intraoperative hemorrhage (4.81% vs 2.85%, *P* = 0.01), postoperative hematoma (3.57% vs 1.68%, *P* = 0.002), post-operative sepsis (13.53% vs 3.85%, *P* < 0.001), peritonitis (11.63% vs 2.9%, *P* < 0.001), percutaneous abdominal drainage (10.85% vs 4.03%, *P* < 0.001) and wound complications (26.71% vs 12.27%, *P* < 0.001). In terms of systemic complications, patients with PEM had higher rates of acute myocardial infarction (1.88% vs 0.46%, *P* = 0.016), pneumonia (6.51% vs 2.28%, *P* = 0.001), tracheostomy (2.48% vs 0.54%, *P* = 0.017), respiratory complications (18.32% vs 8.51%, *P* < 0.001), central nervous system complications (23.72% vs 13.86%, *P* < 0.001), acute kidney injury (16.27% vs 5.73%, *P* < 0.001), total parenteral nutrition (22.48% vs 8.24%, *P* < 0.001), and pressure ulcer (3.26% vs 0.54%, *P* < 0.001).

### Trend study

**Fig. 3:**
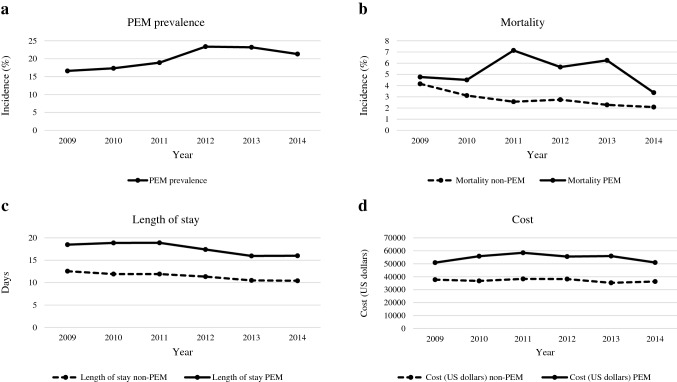
Results of trend on in-hospital outcomes from 2009 to 2014. **a** Trend of PEM prevalence; **b** Trend of the mortality for patients with and without PEM; **c** Trend of length of stay for patients with and without PEM; **d** Trend of cost for patients with and without PEM

Among patients with pancreatic cancer undergoing OPD, the proportion of those with PEM increased from 2009 to 2012 and decreased slightly from 2012 to 2014 (Fig. 3a). The mortality saw a steady decrease in the non-PEM group (from 4.16% to 2.08%). In the meantime, the mortality of patients in the PEM group fluctuated over the 5 years, but the overall trend decreased (from 4.77% to 3.37%, Fig. 3b). Similarly, the length of hospital stay had been steadily decreasing in both the non-PEM group (from 12.6 to 10.4 days) and the PEM group (from 18.5 to 16.0 days, Fig. 3c). Interestingly, the cost of hospitalization remained approximately the same across the 5 years in both groups (37,747.8 USD to 36,287.1 USD in the non-PEM group and 50,881.9 USD to 50,993.0 USD in the PEM group, Fig. 3).

## Discussion

Pancreaticoduodenectomy is a standard surgical procedure to treat pancreatic head tumors. Common complications of PD include pancreatic fistula, biliary fistula, stomach-duodenal fistula, intestinal fistula, GI hemorrhage, cardiac complications, respiratory complications, post-operative sepsis, shock, and wound complications [18, 19]. PEM is prevalent in surgical patients, especially patients with malignant tumors: the prevalence of PEM in general hospitalized patients ranges from 11 to 44% [20]. In our study, 23.0% of all patients who were hospitalized for pancreatic cancer were diagnosed with PEM, which was inconsistent with previous studies. Although there are differences between the scales used to define PEM, here it was defined based on the ICD-9-CM codes which included kwashiorkor, marasmus, cachexia, and other PEM (severe, unspecified) [21]. To the best of our knowledge, this is the first population-based study investigating the impact of PEM on the outcomes of patients with pancreatic cancer undergoing OPD.

Our study showed that PEM is associated with poor perioperative prognosis. After a multivariate analysis, adjusted for the demographic characteristics of patients, socio-economic factors, hospital characteristics and comorbidities, we concluded that PEM is associated with increased mortality, in-hospital adverse events (including surgery-related and systemic), longer LOS, and greater hospital cost.

The in-hospital mortality rate was 3.17% in our study, which was similar to the previous report by Kneuertz et al*.* [22]. The mortality of patients in the PEM group was 2.25 times that of the non-PEM group. A potential explanation is that PEM is associated with an increased incidence of perioperative complications. The trend analysis showed that the mortality of the PEM group was higher than the non-PEM group from 2009 to 2014. In addition, the mortality of the non-PEM group has been steadily decreasing year by year, and the mortality of the PEM group showed a fluctuating decrease, which may be related to the advancement of surgical techniques and pharmacological treatments.

There was a significant difference in hospital stay between the two groups. The hospital cost was also significantly higher in the PEM group than non-PEM group. We also conducted a trend study which indicated that the length of hospital stay was gradually shortening in both groups, and the total hospital cost remained basically unchanged from 2009 to 2014. This may also be due to an improvement in efficiency caused by advances in surgical techniques and pharmacological treatments.

Gastroparesis is a less serious complication of PD and the mechanism is not yet fully understood. Although gastroparesis is not life-threatening, it can prolong hospital stay, increase costs, and have a great impact on the quality of life. It has been reported in the literature that the mechanism of gastroparesis is a complex interaction of various factors such as pylorospasm, diminished hormonal stimulation, inflammation and other complications [23]. The incidence of gastroparesis has been reported in the literature to be 24–70% [24]. However, our study found that the incidence of gastroparesis in the PEM group and the non-PEM group was 6.16% and 2.74% respectively, which were significantly lower than previous reports. We speculate that this may be caused by an underestimation in the diagnosis of gastroparesis.

Our study showed that the incidence of small bowel obstruction was significantly higher in the PEM group. The reason may be the proportion of patients diagnosed with PEM who had small bowel obstruction before surgery was higher than that of the non-PEM group. PEM is also associated with intraoperative hemorrhage and postoperative hematoma. Blood transfusion requirement in the PEM group was higher than in the non-PEM group, but no statistical significance was found. Previous studies reported a significant increase in estimated blood loss and transfusion in the malnutrition group in patients with pancreatic head cancer undergoing PD [25]. We speculate that there may be two reasons for the increased hemorrhage. First of all, compared to patients without PEM, patients in the PEM group may have a longer tumor growth time and a more complicated relationship between the tumor and surrounding tissue, leading to increased difficulty of surgery and hemorrhage risk. Furthermore, patients with PEM have a slow postoperative wound recovery, which increases the risk of bleeding.

Katona et al*.* reported that there may be a potential synergistic effect between PEM and infection [26]. Other researchers also believe that PEM is associated with increased mortality and complications in patients with infectious diseases [27]. Our study found that in pancreatic cancer patients undergoing OPD, PEM was associated with a variety of infectious complications, such as post-operative sepsis, peritonitis, percutaneous abdominal drainage, wound complications and pneumonia.

The overall worse outcomes associated with PEM indicate that it may be one of the signs of disease severity in pancreatic cancer patients undergoing PD, which can help surgeons perform risk stratification, treatment decisions and prognosis prediction. Commonly used nutrition screening tools include Nutritional Risk Index (NRI), [28] Subjective Global Assessment (SGA), [29] Malnutrition Universal Screening Tool (MUST) [30], Nutrition Risk Screening-2002 (NRS-2002), [31] and the Short-Form Mini Nutritional Assessment (MNA-SF) [32]. Among them, NRS-2002 is a highly applicable nutritional assessment tool. As early as 2003, the European Society of Parenteral and Enteral Nutrition recommended it as the preferred nutritional screening tool [31].

Nutritional status and complications are two key factors that affect the recovery of patients with pancreatic cancer who underwent OPD. Reasonable nutritional support can help improve the nutritional status of patients and reduce the risk of complications [33]. A position paper of the International Study Group on Pancreatic Surgery (ISGPS) suggested that preoperative nutritional support should be seriously considered if at least one of the following criteria is met: (1) weight loss > 15% within 6 months, (2) Body Mass Index < 18.5 kg/m2, (3) SGA grade C or nutritional risk score > 5, or (4) serum albumin < 30 g/L (with no evidence of hepatic or renal dysfunction) [34]. Therefore, it is particularly important for surgeons to identify malnutrition well in advance of surgery and provide nutritional support treatment for those patients.

There are several advantages of the current study. First of all, using the full sample of Medicare data from 2012 to 2014, we were able to select a large population. In addition, adjusting the multivariate analysis for patient characteristics, hospital characteristics and comorbidities reduced confounding bias, thus minimizing the impact of selection bias. However, our study presented some drawbacks which may limit our conclusions. First, our study was retrospective, and it is inevitable to have the inherent shortcomings of retrospective research since these challenge the interpretation of causality. Second, some important complications such as pancreatic fistula cannot be analyzed, as they do not have corresponding ICD-9 codes. In addition, histological type of tumor, TNM stage, and neoadjuvant chemotherapy are absent from the NIS database. Finally, since follow-up information is absent from the NIS database, further studies are needed to explore the impact of PEM on the long-term prognosis of patients who underwent OPD.

## Conclusions

The incidence of PEM in pancreatic cancer patients undergoing OPD is relatively high. PEM is associated with higher mortality, greater hospitalization cost and longer length of stay. At the same time, patients with PEM have a higher incidence of a variety of important perioperative complications, such as gastroparesis, small bowel obstruction, hemorrhage, infection and systemic complications. Therefore, for pancreatic cancer patients with poor nutritional status, attention should be paid to correcting their nutrition before surgery as an effective means to optimize postoperative results.
